# Mayer-Rokitansky-Kuster-Hauser Syndrome Type II with Fused Kidneys in Pelvic Cavity: A Case Report

**DOI:** 10.31729/jnma.8532

**Published:** 2024-04-30

**Authors:** Suman Paudel, Prerana Singh Rokaha, Pankaj Kafle

**Affiliations:** 1 Department of Radiology, Kathmandu Medical College and Teaching Hospital, Sinamangal, Kathmandu, Nepal; 2 Kathmandu Medical College and Teaching Hospital, Sinamangal, Kathmandu, Nepal

**Keywords:** *fused kidney*, *Mayer-Rokitansky-Kuster-Hauser syndrome*, *mullerian aplasia*, *uterine aplasia*, *vaginal aplasia*

## Abstract

Mayer-Rokitansky-Kuster-Hauser syndrome (MRKH) also known as Mullerian agenesis, is caused by embryologic underdevelopment of the Mullerian duct, with resultant agenesis or atresia of the vagina, uterus, or both. Patients usually present with primary amenorrhea with normal growth and pubertal development. Here we present a case of a 29-year-old woman presented with primary amenorrhea. Secondary sexual characteristics and hormone evaluation were normal. Ultrasound and MRI were conducted and revealed complete absence of uterus, small vaginal canal. Bilateral renal fossa were empty and both the kidneys were located in the pelvic cavity fused to one-another with single renal pelvis giving pancake appearance.

## INTRODUCTION

Mayer-Rokitansky-Küster-Hauser (MRKH) syndrome, also referred to as Müllerian aplasia, is a congenital disorder characterized by aplasia of the uterus and upper part of the vagina in females with normal secondary sex characteristics and a normal female karyotype (46,XX).^1^ It has an incidence of 1 per 5,000 females.^2^ MRKH can be of two types. Type I is isolated form whereas, type II is accompanied by other malformations involving the kidney, skeletal, and vascular systems.^3^ Patients usually present with primary amenorrhea with normal growth and pubertal development.^1^ MRI is the primary imaging modality to evaluate this illness.^4^

## CASE REPORT

A 29 year old woman presented to our institution with a main complaint of primary amenorrhea. No significant history of any gynecological surgery, radiotherapy, or chemotherapy was present. Physical examination revealed normal development of the secondary sexual characteristics with symmetrical tanner stage IV breast. She had normal distribution of axillary and pubic hair. Gynecological examination showed normal development of labia majora and labia minora with no vaginal opening (pinpoint orifice). Bimanual examination was denied by the patient. Body weight and height was 52 kg and 159 cm, respectively. She underwent ultrasound and MRI of abdomen-pelvis as a part of radiological examination findings of which are discussed separately below. Hormonal evaluation (estrogen, progesterone, FSH) was normal and the karyotype test showed 46,XX chromosomes.

**Figure 1.**
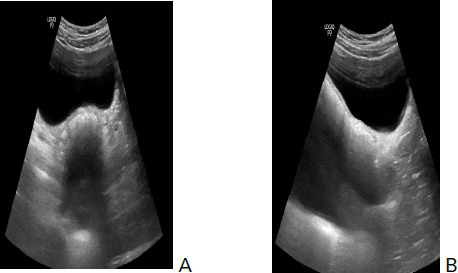
Axial and sagittal ultrasound images showing absence of uterus in pelvic cavity (A) and (B).

Pelvic ultrasound was performed which revealed absence of uterus (Figure 1), empty bilateral renal fossa with both the kidneys fused together located in ectopic position within the pelvic cavity.

**Figure 2.**
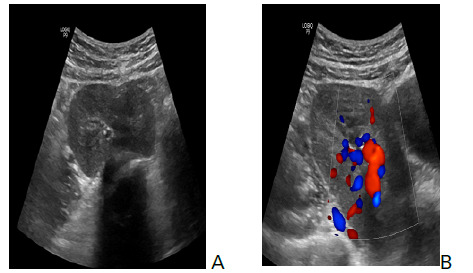
Ultrasound images showing both kidneys fused together within the pelvic cavity (A). Color Doppler examination revealed single renal vessels within the single renal pelvis suggestive of pancake appearance (B).

Doppler examination showed renal vessels within the single renal pelvis suggestive of pancake kidney (Figure 2).

**Figure 3.**
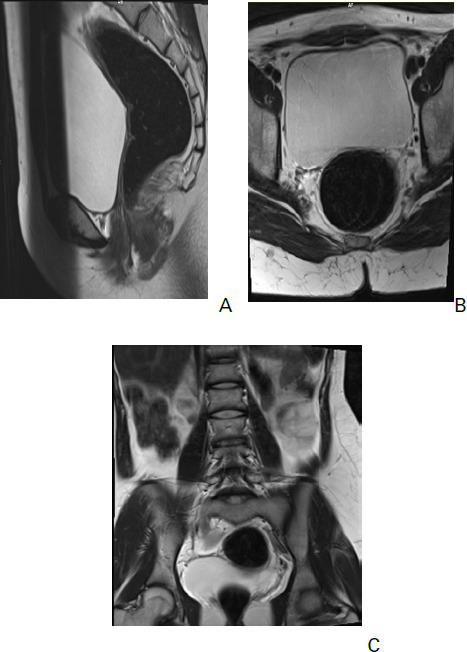
Sagittal and axial T2 weighted MR images show absence of uterus within the pelvic cavity (A) and (B). Coronal T2 weighted MRI shows absence of kidney in bilateral renal fossa (C).

**Figure 4.**
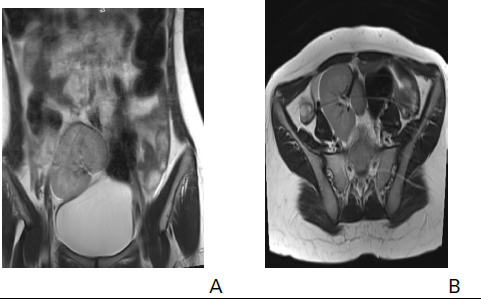
Coronal and axial T2 weighted MRI showing both the kidneys fused together within the pelvic cavity with single renal pelvis giving pancake appearance (A) and (B).

Subsequently, she underwent an MRI of abdomen and pelvis. MRI of the abdomen showed empty bilateral renal fossa, absence of uterus with both kidneys fused together within the pelvic cavity (Figure 3 and 4).

**Figure 5.**
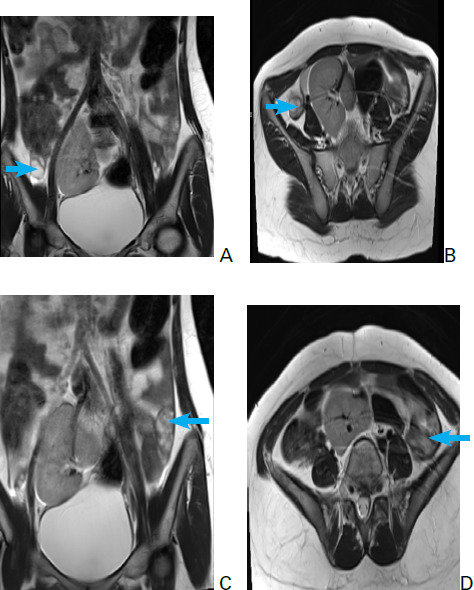
Coronal and axial T2 weighted MR images show the right ovary in more cranial position than normal lateral to the right external iliac artery (A) and (B). Coronal and axial T2 weighted MR images show the left ovary in more cranial position than normal lateral to the left external iliac artery (C) and (D)

Features were suggestive of MRKH syndrome type II (atypical). Bilateral ovaries were normal in size. Both the ovaries were located more cranial than the orthogonal position and lateral to the external iliac arteries suggestive of bilateral ectopic ovaries (Figure 5). Abdominal ultrasound/MRI revealed no significant abnormalities of the liver, gallbladder, pancreas, spleen, and urinary bladder.

Patient was counseled about the diagnosis and prognosis of the condition. No medical/surgical interventions were performed.

## DISCUSSION

There are two types of Mayer-Rokitansky-Kuster-Hauser (MRKH) syndrome with prevalence of type I (56-72%) being more than type II (28-44%) where type I is an isolated form, and type II is accompanied by other malformations involving the kidney, skeletal, and vascular systems, with kidney abnormalities being the most common (30-40%).^1,3-6^ The syndrome is characterized by aplasia of the uterus and upper part of the vagina in females with normal secondary sex characteristics compatible with age, with no sign of virilization and a normal female karyotype (46,XX).^1,4^

Primary amenorrhea is reported, either with or without colic pain, and dyspareunia/apareunia are extra complaints upon referral.^1,4^ Lastly, patients may be referred after unintentionally learning they had uterine or vaginal agenesis; according to reports, the median age of referral was 17.5 years (interquartile range: 1619 years).^1^ In this instance, however, our patient did not exhibit any other clinical symptoms and instead, at the age of 29, came with primary amenorrhea.

Imaging tests, such as ultrasound and magnetic resonance imaging, are required to identify the anatomical features of the condition, whether or not they are associated with laparoscopy. Ultrasonography is frequently used as the first diagnostic test to investigate the presence of ovaries, the absence of a uterus, and kidney abnormalities in cases of type II syndrome.^6^ However, due to technical difficulties, the outcomes can occasionally be unclear.^6^ Magnetic resonance imaging (MRI) is the most sensitive and specific imaging modality for diagnosis confirmation.^4,7-9^ It can also be useful in identifying any associated abnormalities. In MSKH syndrome, ovary anomalies are very uncommon occurring only in 5-10% of the cases whereas in only 16-19% of cases extra-pelvic ovaries are seen but in other case ovaries lie more lateral than normal, lateral to external iliac arteries and uterine remnants found in 48-95% of the cases which increase the risk of endometriosis.^1,6^ In our case, on imaging we found normal ovaries within pelvic cavity lying lateral to external iliac arteries, absent uterus and ectopic kidneys.

This case report illustrates the clinical and radiological findings of Mayer-Rokitansky-Kuster-Hausar syndrome (type II) with associated renal anomalies and bilateral normal ovaries in ectopic location, making it a rare clinical entity.
